# Cell reprogramming using extracellular vesicles from differentiating stem cells into white/beige adipocytes

**DOI:** 10.1126/sciadv.aay6721

**Published:** 2020-03-25

**Authors:** Youn Jae Jung, Hark Kyun Kim, Yoonsuk Cho, Ji Suk Choi, Chang Hee Woo, Kyoung Soo Lee, Jae Hoon Sul, Chan Mi Lee, Jihoon Han, Jae Hyung Park, Dong-Gyu Jo, Yong Woo Cho

**Affiliations:** 1 Department of Materials Science and Chemical Engineering, Hanyang University ERICA, Ansan 15588, Republic of Korea.; 2 ExoStemTech Inc., Ansan 15588, Republic of Korea.; 3 School of Pharmacy, Sungkyunkwan University, Suwon 16419, Republic of Korea.; 4 School of Chemical Engineering, Sungkyunkwan University, Suwon 16419, Republic of Korea.; 5 Biomedical Institute for Convergence, Sungkyunkwan University, Suwon 16419, Republic of Korea.

## Abstract

Stem cell–derived extracellular vesicles (EVs) offer alternative approaches to stem cell–based therapy for regenerative medicine. In this study, stem cell EVs derived during differentiation are developed to use as cell-free therapeutic systems by inducing tissue-specific differentiation. EVs are isolated from human adipose-derived stem cells (HASCs) during white and beige adipogenic differentiation (D-EV and BD-EV, respectively) via tangential flow filtration. D-EV and BD-EV can successfully differentiate HASCs into white and beige adipocytes, respectively. D-EV are transplanted with collagen/methylcellulose hydrogels on the backs of BALB/c mice, and they produce numerous lipid droplets in injected sites. Treatments of BD-EV attenuate diet-induced obesity through browning of adipose tissue in mice. Furthermore, high-fat diet–induced hepatic steatosis and glucose tolerance are improved by BD-EV treatment. miRNAs are responsible for the observed effects of BD-EV. These results reveal that secreted EVs during stem cell differentiation into white adipocytes or beige adipocytes can promote cell reprogramming.

## Teaser

Extracellular vesicles derived during differentiation of stem cells have the potential to induce tissue-specific differentiation.

## INTRODUCTION

Mesenchymal stem cells (MSCs) derived from human tissues such as bone marrow, adipose tissue, and placental tissue are widely clinically used as therapeutic agents for tissue repair and regeneration (*1*, *2*). Human adipose-derived stem cells (HASCs) are the most often used MSCs because of the ease of access by minimally invasive methods and ease of expansion by cell culture (*3*). HASCs are plastic and differentiate into various types of cells, including adipocytes, chondrocytes, osteoblasts, and neurons (*4*). Also, HASCs can be differentiated into functional brown adipocytes by treatment with rosiglitazone (*5*). For these reasons, stem cell–based therapy could be applied to induce lineage-specific tissue regeneration by differentiating tissue cells at injection sites. However, stem cells remain relatively rare after transplantation, about 6% or less of the originally injected cells, and differentiation is inefficient (*6*). These findings suggest that alternative factors from transplanted stem cells promote tissue regeneration by paracrine effects (*7*, *8*).

Previous studies found that these paracrine effects were mediated by extracellular vesicles (EVs) that are secreted by cells (*9*–*11*). EVs, which contain microparticles and exosomes, represent an important role as carriers that contain cytosolic proteins, lipids, and genetic factors such as mRNA, microRNA (miRNA), and noncoding RNA (ncRNA) for intercellular communication (*12*–*16*). Several studies have shown that EVs derived from HASCs provide biochemical cues that are useful for tissue-regenerative medicine and disease treatments (*17*, *18*). Notably, Zhao *et al.* (*19*) demonstrated that exosomes from adipose tissue–derived stem cells attenuated diet-induced obesity (DIO) by inducing polarization of M2 macrophages and beiging of white adipose tissues. Dai *et al.* (*20*) reported that adipose tissue regeneration was effectively induced in 12 weeks after the injection of Matrigel mixed with exosome-like vesicles from adipose tissue. These previous findings suggested that EVs are suitable candidates for use in cell-free therapeutic systems related to adipose tissue regeneration and beiging. In addition, recent reports have shown that EVs obtained during cellular differentiation contain factors such as protein and miRNAs for inducing tissue-specific differentiation (*21*–*25*). Therefore, we hypothesized that EVs derived from differentiating stem cells could be candidates for use in cell-free tissue-regenerative medicine.

All mammals have two main forms of adipocytes, white and brown. White adipocytes play an important role in homeostasis, controlling body temperature and protecting internal organs from external impact. They can also store large amounts of energy by accumulating lipids in the form of triglyceride (*26*). In contrast, brown adipocytes contain many mitochondria and, thus, increase energy expenditure. Brown adipocytes and brown-like adipocytes in white adipose tissues, also called beige adipocytes, have the capacity to dissipate energy. In 2009, brown adipose tissues were identified in human adults by positron emission tomography (*27*). Thus, many researchers became interested in treating obesity and diabetes by activating brown adipose tissues or beige adipoctyes (*28*). Brown adipose tissues are also known to release EVs, which could be biomarkers of brown adipose tissue activity and therapeutic agents for obesity and related diseases (*29*).

In this study, we investigated whether EVs from white/beige adipogenic differentiating HASCs could provide biochemical cues for adipose tissue regeneration and browning. HASC-derived EVs were isolated during white/beige adipogenic differentiation (D-EV and BD-EV, respectively) based on tangential flow filtration (TFF) and characterized. We analyzed miRNAs contained within EVs by next-generation sequencing and identified candidate miRNAs potentially associated with the biological activities of EVs. EVs derived from stem cells during differentiation may be used to functionally enhance adipose tissue through regeneration and transformation.

## RESULTS

### Characterization of EVs

Stem cell–derived EVs have emerged as mediators of cellular function, cell-to-cell communication, and stem cell capacity by conveying bioactive molecules. In particular, recent findings have emphasized the utility of stem cell–derived EVs for tissue regeneration (*30*, *31*). Here, we hypothesized that HASC-derived EVs obtained during white/beige adipogenic differentiation could be used as cell-free therapeutic systems.

**Fig. 1:**
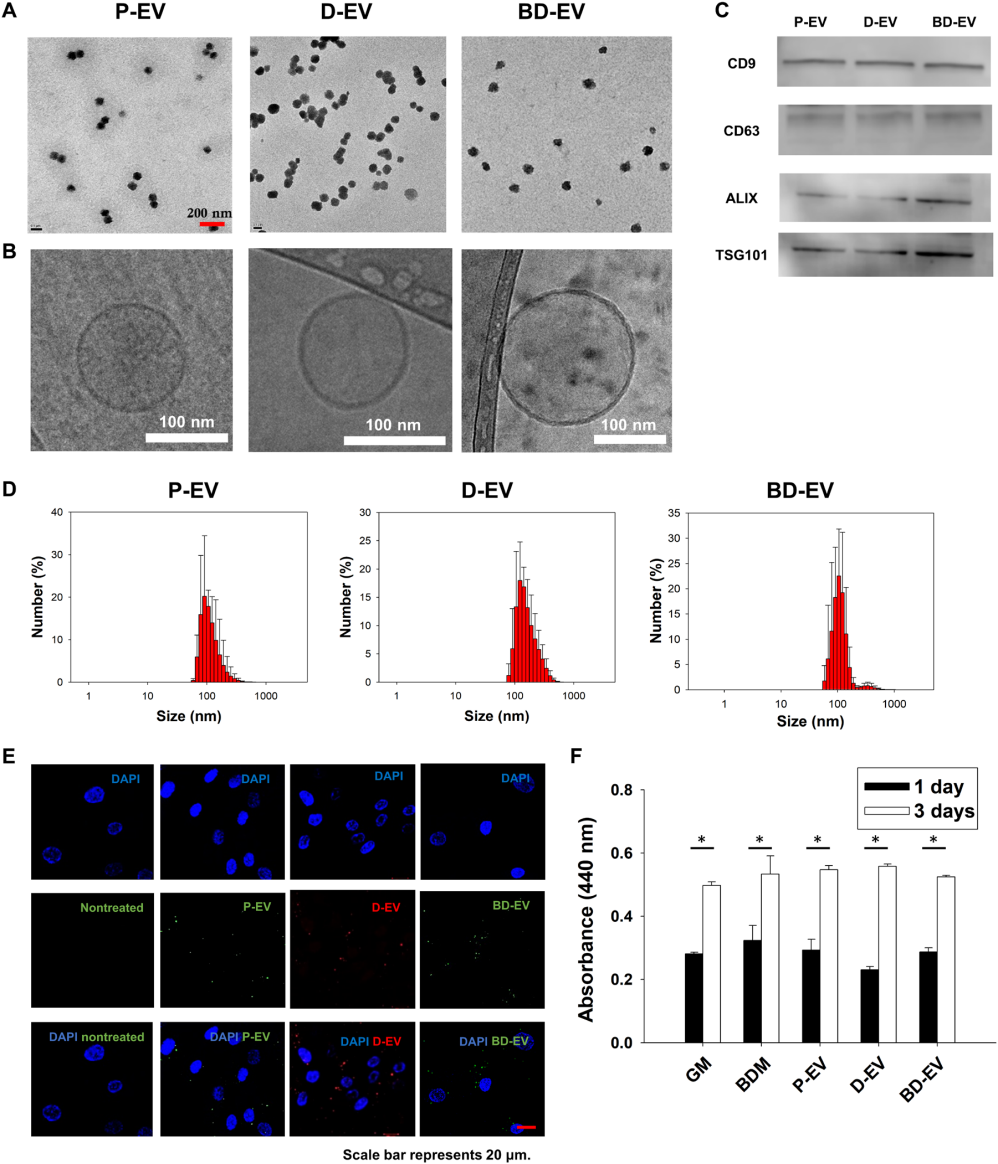
Characterization of HASC-proliferating EVs (P-EV), HASC-white adipogenic differentiating EVs (D-EV), and HASC-beige adipogenic differentiating EVs (BD-EV). (**A**) TEM and (**B**) cryo-TEM images of P-EV, D-EV, and BD-EV. (**C**) Immunoblotting for CD9, CD63, ALIX, and TSG101 of P-EV, D-EV, and BD-EV. (**D**) The size distributions of P-EV, D-EV, and BD-EV were analyzed by dynamic light scattering. (**E**) Confocal laser scanning microscopic images of HASCs after the 3-hour incubation with 1 × 10^8^ particles of DiD-labeled D-EV and PKH26-labeled P-EV and BD-EV. The nontreated group was used as control. Scale bar, 20 μm. (**F**) WST-1–based colorimetric assay to quantify proliferation and viability of HASCs after 1 and 3 days of culture with D-EV and BD-EV (*P < 0.001). Data are shown as the means ± SD from three separate experiments. GM, growth medium; BDM, beige adipogenic differentitation medium.

To produce EVs from D-EV and BD-EV, we cultured HASCs in white/beige adipogenic differentiation medium for 3 weeks. We next examined whether HASCs differentiated into proper white adipocytes or beige adipocytes. Although the morphology of white adipocytes did not differ from that of beige adipocytes, white adipocytes expressed classical adipogenic markers [peroxisome proliferator–activated receptor γ (PPARγ), fatty acid binding protein 4 (FABP4), and Leptin] at slightly different levels of beige adipocytes (fig. S1, A to D). However, the level of UCP1 was markedly increased only in beige adipocytes with an increase in mitochondria (fig. S1, E to G). We isolated D-EV and BD-EV from 500 ml of conditioned medium (CM) collected during differentiation. We also isolated EVs from HASCs during proliferation (P-EV) for isotype control. After purification of EVs using TFF, EVs were characterized by nanoparticle tracking analysis, transmission electron microscopy (TEM), and cryo-TEM. We obtained approximately 1 × 10^10^ to 2 × 10^10^ particles/ml in 10 to 12 ml of volume. As expected, P-EV, D-EV, and BD-EV displayed round vesicles and had diameters from 80 to 120 nm (Fig. 1, A, B, and D). Western blot analyses for exosomal markers showed the presence of CD9, CD63, ALIX, and TSG101 in the purified EVs (Fig. 1C). To determine whether EVs can be internalized in HASCs, the cultured HASCs were treated with 1 × 10^8^ particles/ml of DiD-labeled D-EV or PKH26-labeled P-EV and BD-EV for 3 hours. Next, we examined the internalization of the EVs into the HASCs. P-EV, D-EV, and BD-EV were successfully delivered into HASCs intact (Fig. 1E). We compared the viability of HASCs treated with EVs with vehicle-treated HASCs (growth medium) for 24 and 72 hours. D-EV or BD-EV treatment did not show cytotoxicity in WST-1 assay (Fig. 1F).

### Adipogenesis-related adipokine composition in D-EV and BD-EV

**Fig. 2:**
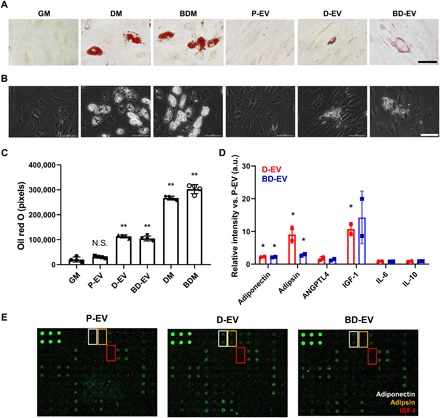
Adipogenic capacity of D-EV and BD-EV. (**A**) Oil red O–stained and (**B**) phase-contrast microscopic images of HASCs treated with 1 × 10^9^ particles/ml of P-EV, D-EV, and BD-EV for 2 weeks. Differentiation medium (DM) and growth medium were used as positive control and negative controls, respectively. Scale bars, 100 μm. (**C**) Oil red O intensity of lipid droplets in each group. All values are expressed as the means ± SD of four independent trials (**P < 0.0001). (**D**) Adipokines within P-EV versus D-EV and BD-EV measured using adipokine array (*P < 0.05). Both down-regulated and up-regulated adipokines were identified in each EV. (**E**) Fluorescent plots for adipokine array. N.S., not significant; a.u., arbitrary units; ANGPTL4, angiopoietin-like 4.

We detected adipokine compositions in P-EV, D-EV, and BD-EV using human adipokine antibody arrays. It has been reported that EVs can efficiently deliver proteins and nucleic acids between cells (*32*, *33*). In addition, lineage-specific EVs contain transcription factors in the form of RNA and protein, which may regulate stem cell fate (*23*). Therefore, we hypothesized that stem cell–derived EVs obtained during specific cellular differentiation processes might contain numerous factors for cellular differentiation. For examination of the adipogenic potentials of D-EV and BD-EV, we incorporated D-EV and BD-EV into culture medium and incubated them with HASCs for 2 weeks. Oil red O–stained and phase-contrast microscopic images showed that lipid droplets were significantly accumulated by D-EV or BD-EV treatment (Fig. 2, A and B). The intensity of Oil red O–stained lipid droplets was increased in D-EV– and BD-EV–treated groups (3.5-fold) compared with the P-EV–treated group (Fig. 2C). The expression levels of several mRNAs among 84 human adipogenesis-related mRNAs also up-regulated in D-EV– and BD-EV–treated HASCs (fig. S2). Notably, the treatment of BD-EV significantly promoted UCP1 expression compared with D-EV–treated HASCs (fig. S3). Several adipokines showed higher expressions in D-EV and BD-EV than in P-EV such as adiponectin (D-EV: 2.24-fold, BD-EV: 2.14-fold), adipsin (D-EV: 9.07-fold, BD-EV: 2.86-fold), angiopoietin-like 4 (ANGPTL4; D-EV: 1.69-fold, BD-EV: 1.35-fold), and insulin-like growth factor (IGF-1; D-EV: 10.71-fold, BD-EV: 14.29-fold), while interleukin-6 (IL-6) and IL-10 were down-regulated in D-EV (0.89-fold and 0.86-fold, respectively) and BD-EV (0.82-fold and 0.91-fold, respectively) (Fig. 2, D and E). It was previously reported that adiponectin and adipsin promoted adipogenic differentiation and augmented gene expression related to adipogenesis (*34*, *35*). Notably, adipsin, which could contribute to lipid accumulation, is much more strongly expressed in D-EV. This finding suggests that D-EV could be capable of promoting adipogenesis. In addition, IGF-1 efficiently promoted adipogenesis of HASCs and antidiabetic action, including improving glucose homeostasis (*36*, *37*). We hypothesized that these adipokines incorporated in D-EV and BD-EV could induce successful adipogenic differentiation. Some inflammation-related adipokines, IL-6 and IL-10, were also expressed in D-EV and BD-EV, although levels were not significantly higher than those in P-EV. In these results, D-EV and BD-EV can be delivered into recipient cells efficiently, where they promote white and beige adipogenic differentiation, although the exact mechanism for adipogenic differentiation remains to be determined.

### In vivo adipose tissue regeneration induced by D-EV

**Fig. 3:**
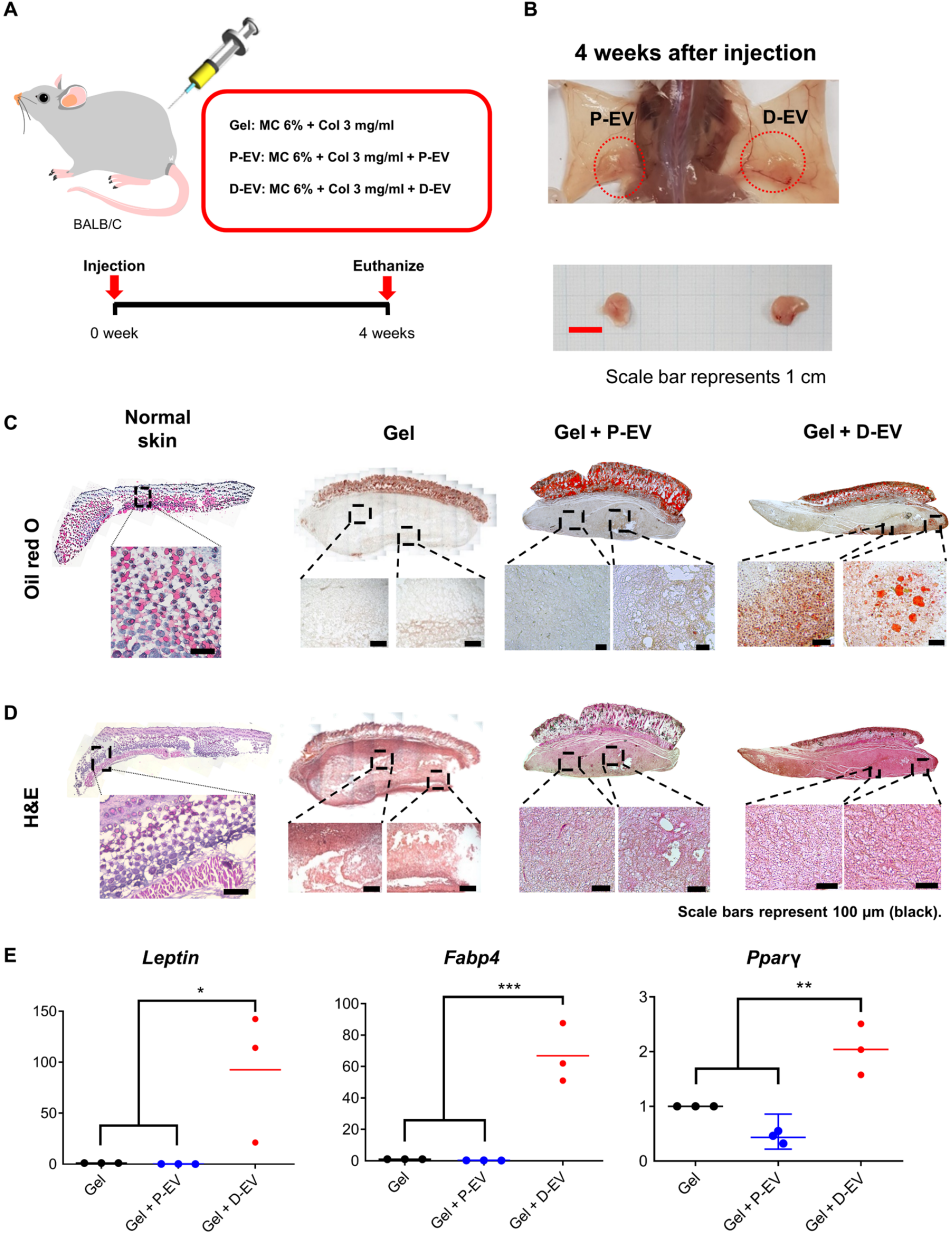
In vivo adipose tissue regeneration using D-EV–containing hydrogels. (**A**) Schematic illustration of in vivo hydrogel injection into the back of each BALB/c mouse (6 weeks old, *n* = 9). (**B**) At 4 weeks after subcutaneous injection, the mice were euthanized, and the grafts were explanted. Scale bar, 1 cm. (Photo credit: Youn Jae Jung and Kyoung Soo Lee, ExoStemTech Inc. and Hanyang University ERICA.) (**C**) Histological evaluation of the grafts and normal mouse skin tissues stained by Oil red O at 4 weeks after injections. Scale bars, 100 μm. (**D**) Histological evaluation of the grafts and normal mouse skin tissues stained by H&E staining. Scale bars, 100 μm. (**E**) qRT-PCR analysis for relative mRNA expression of adipogenic *Leptin*, *Fabp4*, and *Pparg*. Data are shown as means ± SD from three separate experiments (*P < 0.05; **P < 0.01; ***P < 0.001).

To evaluate the effects of D-EV on adipose tissue regeneration in vivo, we subcutaneously injected collagen/methylcellulose (MC) hydrogels with D-EV into BALB/c mice (Fig. 3, A and B), while negative controls were tested with hydrogels alone (Gel) only or P-EV–containing hydrogels. To provide mechanically appropriate conditions and volume-stable adipose tissue formation, we used collagen-MC hydrogels to assess in vivo injection with D-EV because hydrogel systems can support mechanically compatible environments for tissue regeneration, and their stiffness supports cell migration, proliferation, and differentiation through the mechanotransduction pathway (*38*, *39*). We previously reported that soluble extracellular matrix and MC hydrogel enhanced host-derived adipogenesis and angiogenesis without exogenous cells and bioactive molecules throughout 3 weeks (*40*). Similarly, we found that grafts displayed accumulated intracellular lipid droplets 4 weeks after transplantation (Fig. 3C). There were significantly more lipid droplets in grafts of D-EV–loaded hydrogel than in P-EV–loaded hydrogel or hydrogel alone. More host cells had infiltrated both grafts of D-EV– or P-EV–loaded hydrogels compared with hydrogel alone (Fig. 3D). The immunohistology revealed significant increase in the appearance FABP4- and PPARγ-expressing cells and CD163-positive adipose-resident M2 macrophages in grafts of D-EV–loaded hydrogel (fig. S4). These results suggest that D-EV induced migration of endogenous host cells and promoted adipose tissue regeneration in vivo. To evaluate the expression of adipogenesis-related genes in grafts, we performed quantitative polymerase chain reaction (qPCR) analysis. We found that the expression levels of *Leptin*, *Fabp4*, and *Pparg* were significantly higher in the D-EV–loaded group than in the hydrogel-only group or P-EV–loaded group (Fig. 3E). Those genes are related to adipose tissue functions such as lipid transport and storage, glucose metabolism, and homeostasis (*41*, *42*). These results demonstrate that D-EV could contribute to promoting adipogenesis by activating and differentiating host cells.

Although the exact factors for host cell migration and adipogenesis induction remain to be determined, our results suggest that D-EV is a suitable alternative therapeutic agent for adipose tissue regeneration. This EV system can be developed into a cell-free therapeutic system free from the limitations such as tumor formation, contamination by xenogeneic compounds, long-term storage, and large-scale production, which may occur in stem cell–based therapies.

### Adipose tissue browning induced by treatment with BD-EV

**Fig. 4:**
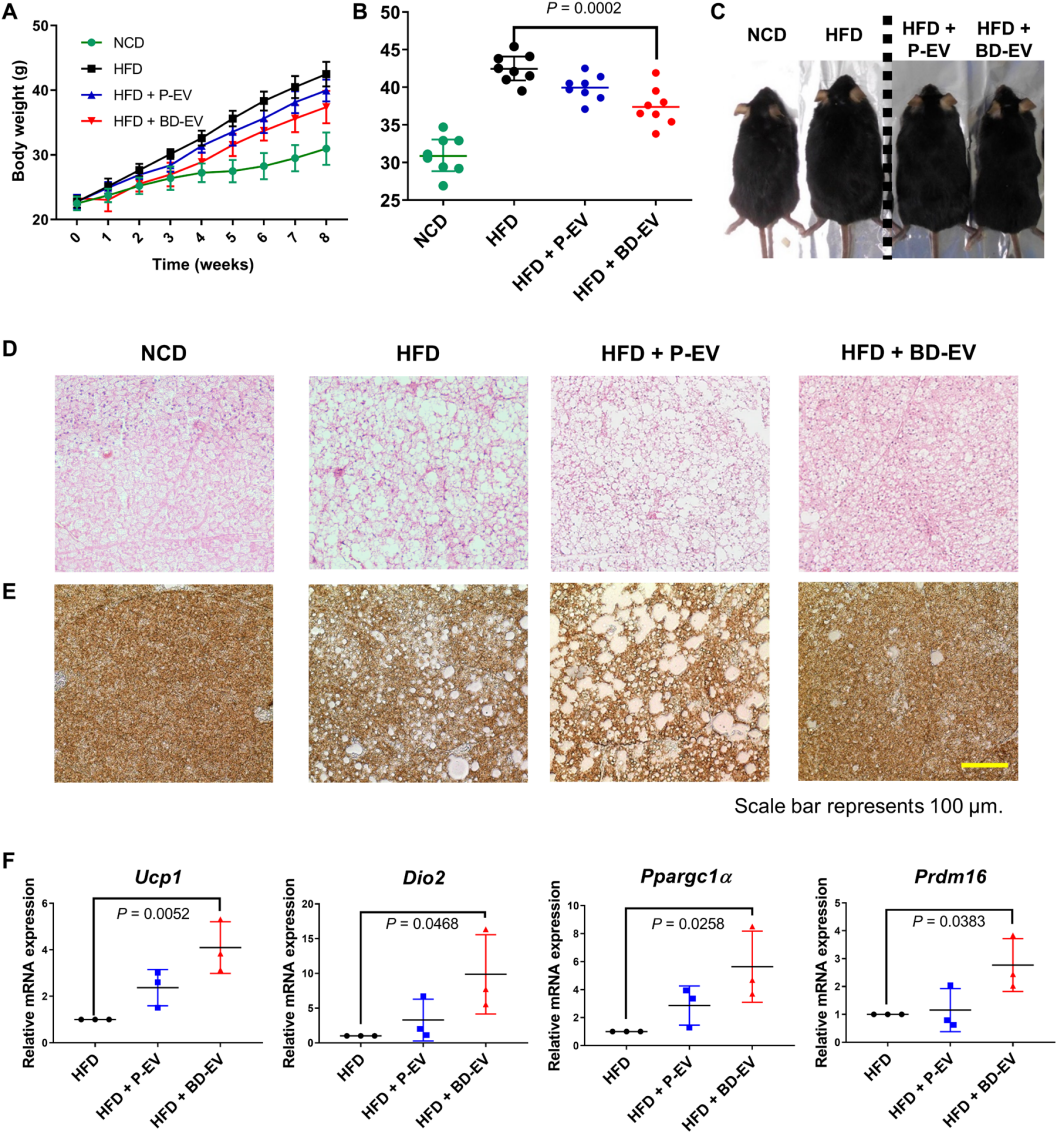
Adipose tissue browning induced by treatment with BD-EV. (**A**) Body weights of C57BL/6 mice at indicated times under high-fat diet (HFD) (*n* = 8 each group). (**B**) Body weights of C57BL/6 mice at 8 weeks after injection. (**C**) Photographs of C57BL/6 mice at 8 weeks after injection. (Photo credit: Youn Jae Jung and Chang Hee Woo, ExoStemTech Inc. and Hanyang University ERICA.) (**D**) Representative images of H&E staining of interscapular brown adipose tissue (iBAT) from 16-week-old HFD-fed mice treated with PBS, P-EV, and BD-EV for 8 weeks. Normal chow diet (NCD)–fed mice used as controls. (**E**) Representative images of sections stained with anti-UCP1 antibody of iBAT from 16-week-old HFD-fed mice treated with PBS, P-EV, and BD-EV for 8 weeks. (**F**) qRT-PCR analysis for relative mRNA expression of adipose tissue browning (*Ucp1*, *Dio2*, *Ppargc1a*, and *Prdm16*). These experiments were performed in triplicate. *P* values are shown in the figures.

To evaluate the effect of BD-EV on DIO, high-fat diet (HFD)–fed C57BL/6 mice were administered twice a week with BD-EV and weight changes were observed for 8 weeks. Mice were intraperitoneally injected with 3 × 10^8^ particles/ml of BD-EV or P-EV for 8 weeks; control mice were injected with vehicle [phosphate-buffered saline (PBS)]. Mice treated with P-EV or PBS showed no notable difference in body weight at the end of the treatment. However, BD-EV–treated mice displayed 22.01% inhibition of weight gain compared with the PBS-treated mice at the end of 8 weeks of treatment (Fig. 4, A to C). This result suggests that BD-EV treatment can ameliorate DIO development.

To better understand the attenuated body weight gain, we assessed the effects of BD-EV on brown adipose tissues of HFD-fed mice. Using hematoxylin and eosin (H&E)–stained brown adipose tissues, we detected that brown adipose tissues were activated in BD-EV–treated mice, although more brown adipose tissues were whitened in PBS-treated mice (Fig. 4D). In the immunohistochemical analysis of brown adipose tissues, the immunoreactivity of *UCP1* was apparently increased in BD-EV–treated mice compared with the PBS-treated mice, indicating that BD-EV–induced *UCP1* promotes brown fat activation (Fig. 4E). To further confirm the browning effect in brown adipose tissues, we performed qPCR to investigate whether genes related to adipose tissue browning were expressed by BD-EV treatment (Fig. 4F). In brown adipose tissues, *Ucp1* expression was significantly up-regulated in BD-EV–treated mice; notably, increased type 2 deiodinase (*Dio2*) expression indicated an active brown fat phenotype, although other related genes were not altered significantly. The expression levels of genes related to brown and beige adipocytes (*Ppargc1a* and *Prdm16*) were also up-regulated in BD-EV–treated mice. These results indicate that BD-EV activated gene expression related to adipose tissue browning of brown adipose tissues. Since BD-EV was produced during the differentiation of HASCs into beige adipocytes by rosiglitazone, the brown fat activation effect of BD-EV may arise from rosiglitazone remaining in BD-EV. Therefore, we investigated whether BD-EV contains rosiglitazone. High-performance liquid chromatography analysis showed no detectable rosiglitazone in BD-EV (fig. S5).

Next, we performed histological analyses of white adipose tissues in HFD-fed mice and found fewer adipocytes in inguinal white adipose tissue in BD-EV–treated mice than in P-EV– or PBS-treated mice, determined by H&E staining and adipocyte area analysis (fig. S6, A and C). BD-EV also induced *Ucp1* expression in white adipose tissues and substantially increased the multilocular adipocytes (fig. S6B). In addition, genes related to adipose tissue browning (*Ucp1*, *Tbx1*, *Epsti1*, *Ppargc1a*, and *Dio2*) were highly expressed in BD-EV–treated mice (fig. S6, C to G). These results indicate that BD-EV can promote brown adipogenesis and browning of white adipocytes.

### Effects of BD-EV on liver steatosis

**Fig. 5:**
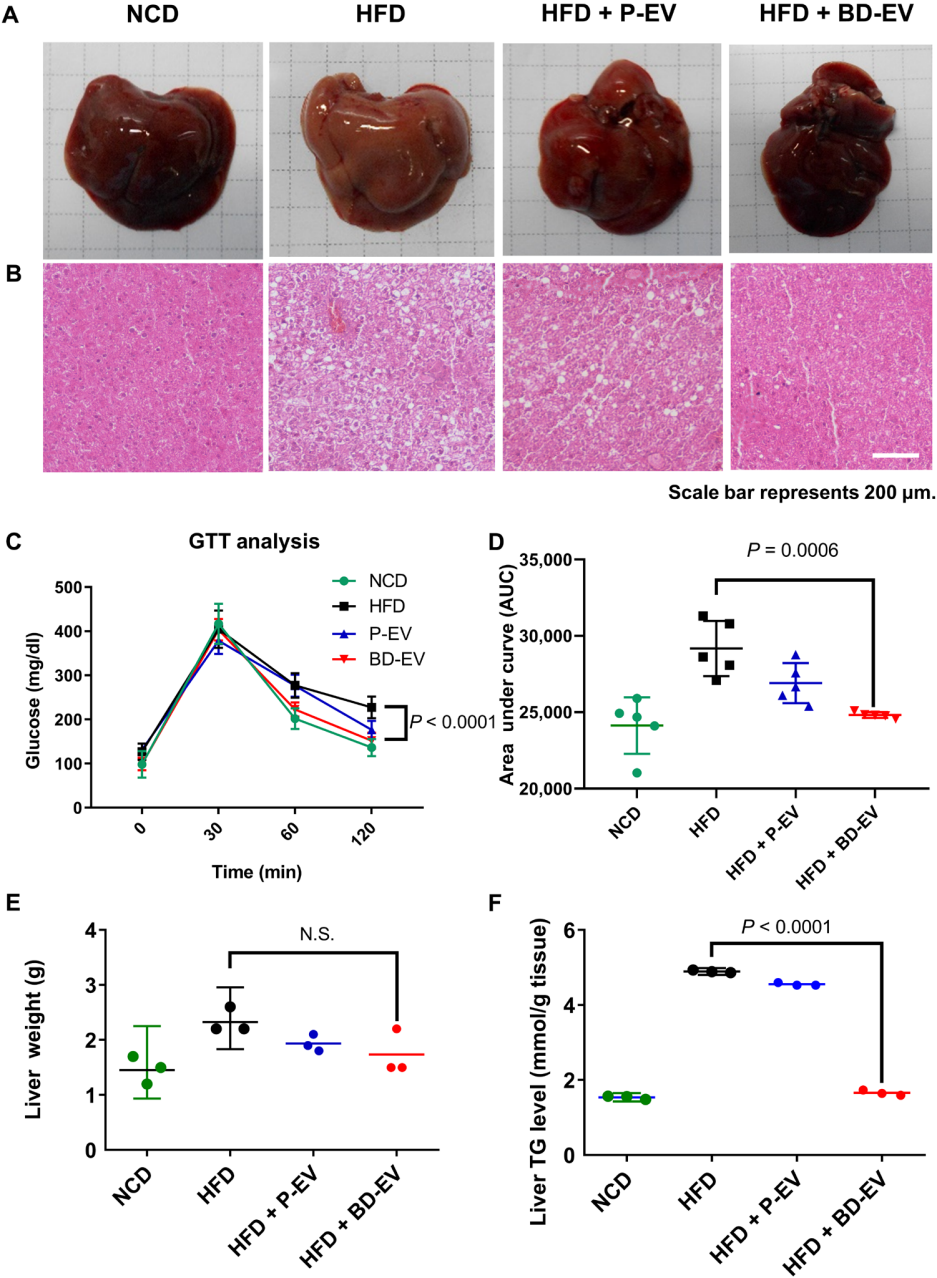
Administration of BD-EV ameliorates hepatic steatosis in HFD-fed mice. Photographs (**A**) and H&E-stained images (**B**) of liver tissue from 16-week-old mice treated with PBS, P-EV, and BD-EV for 8 weeks. NCD-fed mice used as control. (Photo credit: Youn Jae Jung and Chang Hee Woo, ExoStemTech Inc. and Hanyang University ERICA.) (**C**) Glucose tolerance test was performed after 12 hours of fasting of 16-week-old mice. (**D**) Calculated area under curve during glucose tolerance test (*n*=5 per group). (**E**) Quantification of liver tissue weight from 16-week-old mice treated with PBS, P-EV, and BD-EV for 8 weeks. (**F**) Liver triglycerides were quantified from liver tissues from 16-week-old mice treated with PBS, P-EV, and BD-EV for 8 weeks. NCD-treated mice were used as controls (*n* = 3 per group). *P* values are shown in the figures.

Obesity is associated with adipose tissue inflammation that can induce the development of metabolic diseases, including liver steatosis (*43*). DIO promotes hepatic steatosis and accumulates numerous lipid droplets in liver tissue (*44*). We investigated whether BD-EV had any ameliorating effect on liver steatosis. We found that 8-week HFD-fed mice show hepatic steatosis (Fig. 5A). As expected, numerous lipid droplets were present in liver tissues of PBS-treated HFD-fed groups (Fig. 5B). However, the liver tissues in BD-EV–treated HFD-fed mice showed fewer lipid droplets than PBS- and P-EV–treated mice. H&E-stained liver tissue image of BD-EV–administered HFD-fed animal is difficult to distinguish from liver tissue image of normal chow diet (NCD) animal. These results demonstrate that BD-EV treatment prevents lipid accumulation in liver tissue of HFD-fed mouse. For further investigation, we performed the glucose tolerance test to determine whether BD-EV treatment restored glucose metabolism. BD-EV–treated mice showed significantly higher sensitivity to glucose than the other groups (Fig. 5, C and D). Consistently, liver triglyceride levels in HFD-fed mice treated with BD-EV were significantly lower than levels in P-EV– or PBS-treated groups, even though liver weight did not differ significantly across groups (Fig. 5, E and F). These results suggest that BD-EV can effectively prevent hepatic steatosis induced by DIO.

### Analysis of miRNA expression profile in EVs

**Fig. 6:**
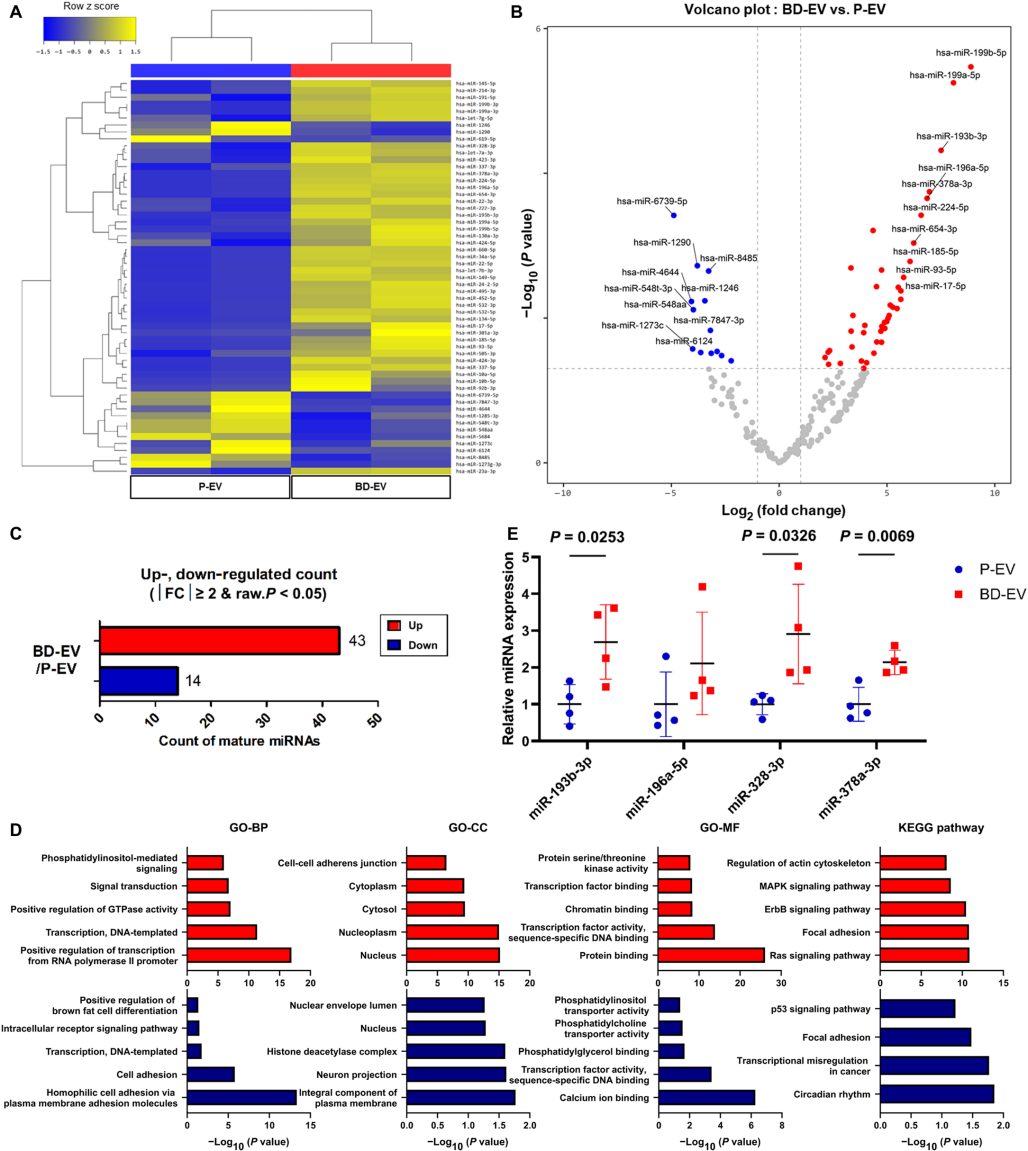
EV miRNA profiling of P-EV and BD-EV. (**A**) Hierarchical cluster analysis of the significantly expressed miRNAs from P-EV and BD-EV. (**B**) Volcano plot of small RNA sequencing–identified miRNAs in BD-EV versus P-EV. (**C**) Numbers of differentially up-regulated and down-regulated count by fold change and *P* value. (**D**) GO and KEGG pathway analyses of the target genes of the up-regulated and down-regulated miRNAs. (**E**) Expression levels of miRNA was confirmed in P-EV and BD-EV using qPCR (*n* = 4). *P* values are shown in the figures. BP, biological processes; CC, cellular components; MF, molecular functions.

Transplantation of brown adipose tissue from normal mice into mice with adipose tissue–specific knockout of the Dicer improved glucose tolerance through exosomal miRNAs (*45*). To profile the EV miRNAs, total RNA was purified from P-EV and BD-EV and used for small RNA sequencing. About 60 million to 80 million of raw reads were obtained using Illumina HiSeq. The raw reads of EV miRNAs were preprocessed, analyzed with miRDeep2, and then trimmed for adapter. A total of 57 significantly differentially expressed miRNAs were identified, and hierarchical clustering showed that the miRNA expression profile of BD-EV was distinct from that of P-EV (Fig. 6, A to C). To further investigate the function of the differentially expressed miRNAs in BD-EV and P-EV, we used the Database for Annotation, Visualization and Integrated Discovery (DAVID) functional annotation tool for performing the Gene Ontology (GO) analysis. The significantly enriched GO terms of biological processes, cellular components, and molecular functions were selectively presented. For the up-regulated miRNAs of BD-EV, “positive regulation of transcription” in the biological process category, “nucleus” and “nucleoplasm” in the cellular component category, and “protein binding” and “sequence-specific DNA binding” in the molecular function category were observed to be significant. For the down-regulated miRNAs of BD-EV, “positive regulation of brown fat cell differentiation” in the biological process category, “integral component of plasma membrane” in the cellular component category, and sequence-specific DNA binding in the molecular function category were significant. Also, we used a Kyoto Encylopedia of Genes and Genomes (KEGG) pathway enrichment analysis to verify signaling pathways in each differentially expressed miRNAs in BD-EV and P-EV. KEGG analysis showed “Ras signaling,” “ErbB signaling pathway” and “MAPK (mitogen-activated protein kinase) signaling” in target genes of up-regulated miRNAs, and “p53 signaling pathway” in target genes of down-regulated miRNAs (Fig. 6D). Among significantly up-regulated miRNAs, miR-193b, miR-196a, miR-328, and miR-378a are reported to play important roles in brown fat differentiation (*46*–*49*). We validated four of the miRNAs that are differentially expressed using qPCR. miR-193b, miR-328, and miR-378a were significantly enriched in BD-EV compared with P-EV. miR-196a showed trends of up-regulation in the BD-EV (Fig. 6E). Collectively, these results indicate that possible miRNAs contributing to brown fat cell differentiation are enriched in BD-EV compared with P-EV.

## DISCUSSION

EVs are membrane vesicles secreted from almost all of the mammalian cells. They contain bioactive proteins and genetic factors such as mRNA, miRNA, and ncRNA for cell-to-cell communication and modulate the molecular activities of recipient cells. EVs derived from MSCs are known to control cellular proliferation and differentiation. Therefore, these EVs have been investigated as a potential tissue-regenerative medicine. However, they have limited efficacy for tissue-specific differentiation in defective tissues, so they still need to be modulated to enhance tissue-specific differentiation.

In this study, we developed EVs from HASCs during white and beige adipogenic differentiation (D-EV and BD-EV, respectively). D-EV and BD-EV contain various factors for differentiation of stem cells into white/beige adipocytes and promote adipogenesis in vitro and in vivo (fig. S7). D-EV promoted adipose tissue regeneration in vivo at 4 weeks after injections, resulting in elevated PPARγ, FABP4, and leptin expression. BD-EV prevented DIO by inducing adipose tissue browning. In addition, BD-EV suppressed the accumulation of lipid droplets in liver tissue and restored glucose homeostasis. We propose that these EVs could serve as cell-free therapeutic systems.

We obtained and characterized EVs secreted from stem cells during white or beige adipogenic differentiation for use in cell-free therapeutic systems. HASCs were isolated from adipose tissue of human adults. Therefore, negative effects from donor variables, such as age, gender, health status, and the fat origin of HASCs from different fat depots, could be present. To reduce these variables of HASC characteristics, the International Society for Cellular Therapy (ISCT) proposed minimal criteria to define human MSCs (*50*). Therefore, we purchased clinically applicable HASCs that meet minimal quality criteria stated by the ISCT from Cefo Bio Co. Ltd. (Seoul, Korea). For repeating tests and further studies, especially preclinical and clinical applications, a large number of HASCs are required and should be banked (*51*, *52*). Consequently, we developed stem cell banking to store large amount of cells and to use equivalent cells in this study.

The HASCs converted by D-EV exhibited morphological and molecular properties of white adipocytes and beige adipocytes. D-EV promoted adipogenic differentiation both in vitro and in vivo. D-EV adipogenicity in vitro was less effective than in vivo because EVs may not only regulate cell-to-cell communication but also modulate tissue microenvironment indirectly (*53*). We report here that D-EV contain various adipokines, such as adiponectin and adipsin, which are efficient components of adipose tissue regeneration by inducing direct adipogenic differentiation. FABP4 highly positive cells were significantly observed in D-EV–containing hydrogel, although PPARγ level was only slightly detected. These findings correlated with mRNA expression results. Adipose tissue regeneration was also induced by infiltration of adipose-resident M2 macrophages. This is in line with the previously reported in situ adipose tissue regeneration (*40*). Although this study suggested that D-EV induce adipose tissue regeneration efficiently, further mechanistic studies, such as immune microenvironment for regeneration, need to be carried out.

Next, we found that BD-EV prevented DIO by inducing adipose tissue browning both in brown and white adipose tissues. According to previous studies, both activation of brown adipose tissue and browning of white adipose tissue are described as increased expression levels of UCP1 in brown/white adipose tissues and multilocular adipocytes (*54*, *55*). *Ucp1*-positive adipocytes were found more in brown adipose tissues and inguinal white adipose tissues from BD-EV–treated mice compared with P-EV–treated mice. Several genes related to adipose tissue browning were highly expressed in both tissues. Together, these findings propose that BD-EV treatments increase the amount of *Ucp1* both in brown and white adipose tissues, and those were correlated with adipose tissue browning. Moreover, BD-EV suppressed the accumulation of lipid droplets in liver tissue and restored glucose homeostasis in HFD-fed mice. We found that candidate miRNAs might be responsible for the differentiating effects of BD-EV. The recent study reported that numerous miRNA are present in adipose tissues, actively participating in the regulation of adipogenesis, adipokine secretion, inflammation, and intercellular communications in the local tissues (*56*). In conclusion, the fate of stem cells can be efficiently manipulated using EVs obtained from differentiating stem cells, and these differentiating EVs can be used as a remedy to regenerate or reprogram many tissues.

Stem cells have the plasticity to differentiate into various types of cells, so stem cell–based therapy has been applied for tissue-regenerative medicine. However, given the recent studies of stem cell therapy, alternative approaches are necessary because of their limitations, such as inefficient differentiation or low viability of injected cells. Our finding that EVs during white/beige adipogenic differentiation induce cellular differentiation suggests a potential for cell-free therapeutic systems by inducing tissue-specific differentiation in defective tissues.

## MATERIALS AND METHODS

### Cell culture

We purchased HASCs (primary MSCs) from Cefo Bio Co. Ltd., and then HASCs were stocked as master cell bank (MCB) at passage 0 (table S1). HASCs were cultured using Dulbecco’s modified Eagle’s medium (DMEM) supplemented with 10% fetal bovine serum (FBS) and penicillin/streptomycin (P/S; 1000 UI/ml) at 37°C in humidified air containing 5% CO_2_. The cultured HASCs were stocked as working cell bank (WCB) at passage 3. Then, HASCs at passage 3 were cultured, and we replaced the medium every 3 to 4 days with fresh media, and HASCs were further expanded by plating 1 × 10^6^ at passage 5. Cells were subcultured with 0.05% trypsin-EDTA. To differentiate white adipogenesis, we maintained HASCs at passage 5 in DMEM supplemented with 5% FBS, P/S (1000 UI/ml), 1 μM dexamethasone, 0.5 mM 3-isobutyl-1-methylxanthine (IBMX), insulin (10 μg/ml), and 100 μM indomethacin. To induce beige adipocyte differentiation, we maintained HASCs in DMEM supplemented with 5% FBS, P/S (1000 UI/ml), 1 μM dexamethasone, 0.5 mM IBMX, insulin (10 μg/ml), and 2 μM rosiglitazone. We also changed this medium every 3 days for 14 days.

### Isolation and characterization of P-EV, D-EV, and BD-EV

To prepare CM for production of EVs, we collected cell culture medium during white/beige adipogenic differentiation of HASCs and replaced it with serum-free medium for 24 hours. We isolated D-EV and BD-EV from CM using a previously reported method with slight modifications (*57*). Briefly, we centrifuged CM at 300*g* for 5 min to eliminate cellular debris. Subsequently, we removed the soluble proteins from the supernatant of CM by TFF using a 0.22-μm bottle top filter (Millipore, Billerica, MA, USA) and 300-kDa MWCO (molecular weight cutoff) ultrafiltration membrane filter capsule (Pall Corporation, Port Washington, NY, USA). We performed continuous circulation at 4 ml/min operation speed to remove the contaminants below 500 kDa and obtained EVs in a final volume of approximately 10 ml. We quantified EVs using a micro bicinchoninic acid protein assay (Thermo Fisher Scientific, Rockford, IL, USA) and nanoparticle tracking analysis (LM-10, Malvern Instruments Ltd, UK) equipped with 642-nm laser module at room temperature. We introduced samples into the chamber manually and recorded video images for 30 s. We performed each experiment out in triplicate. We measured the size distributions of the EVs by dynamic light scattering (Zetasizer ZS90, Malvern Instruments Ltd.). We used P-EV isolated during HASC maintenance (passages 5 to 7) as an isotype control of D-EV and BD-EV. We performed immunoblotting to identify the presence of exosomal markers CD9 (ab223052, Abcam; 1:1000 dilution), CD63 (ab68418, Abcam; 1:1000 dilution), ALIX (ab76608, Abcam; 1:1000 dilution), and TSG101 (ab30871, Abcam; 1:1000 dilution) in EVs using protein extracted from EVs at 4°C for 24 hours and goat anti-rabbit secondary antibody (ab6721, Abcam; 1:100 dilution) at room temperature for 2 hours.

### Transmission electron microscopy

To visualize the morphology of P-EV, D-EV, and BD-EV, we fixed samples with 0.5% glutaraldehyde solution overnight for TEM. Subsequently, we centrifuged the samples at 13,000*g* for 3 min and dehydrated the pellets in absolute ethanol for 10 min and then dropped them onto formvar-carbon–coated copper grids (Ted Pella Inc., Redding, CA, USA). We stained the samples with 1% phosphotungstic acid for 1 min and washed them with absolute ethanol. We stored the grids in a desiccator before analysis and then observed the samples on a JEM-2100F field emission electron microscope (JEOL Ltd., Japan). For cryo-TEM analysis, P-EV, D-EV, and BD-EV were collected on lacey carbon grid (Electron Microscopy Sciences, Hatfield, PA, USA). The grids were stored in liquid nitrogen and then transferred to a cryo-specimen holder and maintained at −180°C. Images were collected on the Tecnai Twin transmission electron microscope operating at 200 kV.

### Cellular uptake of EVs

We seeded 1 × 10^5^ HASCs at passage 5 per well in a confocal dish (catalog no. 200350, SPL). For DiD-labeled D-EV treatments, we labeled D-EV with DiD for 30 min at 37°C. For PKH26-labeled P-EV and BD-EV treatments, we labeled P-EV and BD-EV with PKH26 for 30 s at room temperature. After incubation, we removed unreacted DiD and PKH26 with a column (MWCO 3000 Da, Invitrogen, CA). We exposed HASCs to 1 × 10^8^ particles/ml of DiD-labeled D-EV and PKH26-labeled P-EV and BD-EV for 3 hours. We stained the nuclei with 4′,6-diamidino-2-phenylindole (DAPI). The nontreated group was used as negative control. The HASCs were visualized by confocal laser scanning microscopy (Zeiss, Weimar, Germany).

### Cell viability assay

We seeded HASCs at 1 × 10^4^ cells per well into 48-well plates in DMEM containing 10% FBS and 1% P/S and incubated these overnight at 37°C under 5% CO_2_. We washed the cells with PBS and then changed the medium to growth medium (DMEM containing 5% FBS and 1% P/S), beige adipogenic differentiation medium, and medium containing EVs (P-EV, D-EV, and BD-EV with 1 × 10^8^ particles/ml). After incubating for 24 and 72 hours, we washed the cells with PBS and added WST-1 solution (Roche, Mannheim, Germany) mixed medium (1:10, WST-1:media) to each well. We incubated the plates for 1 hour at 37°C under 5% CO_2_. We then measured the absorbance at 440 nm using a microplate spectrophotometer (PowerWave XS, BioTek Instruments, Winooski, VT, USA, www.biotek.com). We performed these experiments in triplicate.

### Adipokine antibody array

We analyzed adipokines in P-EV, D-EV, and BD-EV using a human adipokine antibody array kit (RayBiotech, Norcross, GA, USA) according to the manufacturer’s protocol. The array glass chip containing 62 different human adipokine antibodies was blocked and incubated with lysate of EVs. The glass chip was washed and subsequently treated with biotin-conjugated antibodies. After incubation with fluorescent dye–conjugated streptavidin, we detected cytokine signals by a laser scanner (Molecular Devices, Sunnyvale, CA, USA, www.moleculardevices.com) using the Cy3 channel. We quantified signal intensities with GenePix Pro software (Molecular Devices, CA, USA).

### Oil red O staining

After adipogenic induction using growth, adipogenic or beige adipogenic differentiation media, P-EV, D-EV, BD-EV, and IGF-1, we fixed cells in 4% paraformaldehyde for 30 min, washed them with 60% isopropanol, and stained them with Oil red O solution for 10 min at room temperature, followed by repeated washing with tap water. Stained cells were observed by light microscopy (KI-2000, Korea Labtech Corporation, Korea).

### Adipose tissue regeneration in animal models

We assessed the efficacy of EVs for adipose tissue regeneration using mouse models. We performed all experiments with approval from the institutional review board of Hanyang University (IRB number HYI-15-209-1). We prepared MC and collagen-based injectable hydrogels to deliver the EVs into the subcutaneous tissues of mice. MC (6 weight %) and type 1 rat tail collagen (3 mg/ml; BD Biosciences, Bedford, MA, USA) solutions were mixed and homogeneously stirred at 4°C. We subcutaneously injected 250 μl of the collagen/MC pre-gel solutions with and without P-EV or D-EV (3 × 10^8^ particles/ml) into the backs of male BALB/c mice (6 weeks old) using an 18-gauge needle. After 4 weeks postinjection, we explanted the grafts and immediately fixed them in 4% paraformaldehyde for further analysis. We analyzed nine mice per experimental group. We isolated total RNA from hydrogel and hydrogel loaded with P-EV and D-EV using easy-Blue (17061, Intronbio, MA, USA). We performed reverse transcription of 0.5 μg of total RNA using RT2 First Strand kit (Qiagen, Hilden, Germany) and performed qPCR with SYBR Green Master Mix (Qiagen). Primers that target *Leptin*, *Fabp4*, *Ppar*γ, and *Actb* were purchased commercially (Qiagen). We calculated relative mRNA gene expressions using *Actb* as a control and the 2^−Δct^ method.

### Histological analysis of explanted tissues

For histological evaluation, we fixed the grafts in 4% paraformaldehyde, embedded them in optimal cutting temperature compound, and froze them at −70°C. We sliced the frozen samples into 5-μm sections using a cryostat and processed them for H&E staining and Oil red O staining.

### High fat–fed animal models and diet

Male C57BL/6 mice were purchased from Orient Bio (Sungnam, Korea) and housed individually in a temperature-controlled room with a 12-hour light/dark cycle. After a 1-week adaption period, at age 8 weeks, the mice were fed HFD for 8 weeks. We used mice fed on NCD as controls. We purchased the high-fat (60% kcal from fat) food from Central Lab Animal Inc. (Seoul, Korea). We performed all experiments with approval from the institutional review board of Hanyang University.

### Intraperitoneal administration of BD-EV

During HFD feeding, we intraperitoneally administered BD-EV (3 × 10^8^ particles per mouse, twice a week) and P-EV (3 × 10^8^ particles per mouse, twice a week). We used HFD- or NCD-fed mice treated with PBS as controls. We measured body weight and food intake during administration. After the intervention, mice were euthanized under anesthesia with CO_2_ gas.

### RNA isolation from adipose tissues and qPCR

We isolated total RNA from brown adipose tissues treated with PBS, P-EV, and BD-EV using easy-Blue (17061, Intronbio, MA, USA). We performed reverse transcription of 0.5 μg of total RNA using RT2 First Strand kit (Qiagen) and performed qPCR with SYBR Green Master Mix (Qiagen). Primers that target *Ucp1*, *Dio2*, *Ppargc1a*, *Prdm16*, and *Actb* were purchased commercially (Qiagen). We calculated relative mRNA gene expressions using Actb as a control and the 2^−Δct^ method. To isolate total RNA from inguinal white adipose tissues, we collected RNA from inguinal white adipose tissues of 16-week-old HFD-fed mice following a published protocol with minor modifications (*58*). Briefly, we freshly dissected about 100 mg of tissue and homogenized it in 1 ml of RNAiso PLUS (Takara Bio). We centrifuged the samples at 12,000*g* at 4°C for 10 min to obtain fat monolayer. Carefully avoiding this layer, we pipetted the remaining solution that contained RNA into tubes. We performed the next step in RNA isolation as described previously (*59*).

### Glucose tolerance test

We performed the glucose tolerance test after 8 weeks of intervention. After 12 hours of fasting, we intraperitoneally injected glucose solution in mice (2 g/kg body weight). We measured blood glucose levels at different time points using Accu-Chek (Roche, Switzerland).

### Liver triglyceride quantification

We quantified the lipids we extracted from the liver tissues and hepatic TG with a triglyceride detection kit (ab102513, Abcam, MA, USA). We normalized total hepatic TG to liver wet weight.

### Histological and immunohistochemical analyses (adipose tissue formation)

For histological evaluation of adipose tissue formation, we fixed the grafts in 4% paraformaldehyde and embedded them in paraffin or optimal cutting temperature (OCT) compound. The OCT compound–embedded samples were frozen at –70°C. The paraffin-embedded samples and frozen samples were sliced into 5-μm sections using a cryostat and processed them for H&E staining and Oil red O staining. Anti-CD163 (sc-58965; 1:00; Santa Cruz Biotechnology) was stained for adipose-resident M2 macrophages. Anti-FABP4 (2120s; 1:100; Cell Signaling Technology) and anti-PPARγ (sc7273; 1:100; Santa Cruz Biotechnology) were stained for adipocytes. Horseradish peroxidase (HRP)–conjugated secondary antibody was used (ab97051; 1:1000; Abcam), and samples were stained using DAB staining kit [rabbit-specific HRP/DAB (ABC) detection immunohistochemistry kit, ab64261, Abcam].

### Immunohistochemistry

We sectioned the paraffin-embedded tissues into 5-μm slides and deparaffinized them by placing them in an oven at 60°C for 10 min. We stained the slides with a UCP1 antibody (ab10983, Abcam; 1:500 dilution) at 4°C overnight and with goat anti-rabbit secondary antibody (ab6721, Abcam; 1:1000 dilution) at room temperature for 2 hours. We mounted samples with mounting medium (Permount, Fisher Scientific, UK).

### Small RNA sequencing and data analysis

RNA extraction from EVs, library preparation, cluster generation, and sequencing were performed by Macrogen (Seoul, Korea). Briefly, EV RNA was extracted using the Maxwell RSC miRNA from plasma (Promega) according to the manufacturer’s instructions. RNA (10 ng) from EVs is polyadenylated to provide a priming sequence for an oligo (dT) primer, and then Illumina adapters are added. Complementary DNA was synthesized, and PCR amplification was performed. Libraries for small RNA were constructed using SMARTer smRNA-Seq Kit for Illumina (Takara Bio, Shiga, Japan) according to the manufacturer’s instructions. We gel purified the libraries and validated them on the Agilent Bioanalyzer (Agilent Technologies, Waldbronn, Germany). The libraries were sequenced on an Illumina HiSeq 2500 instrument (Illumina, San Diego, CA). EV miRNA target genes were predicted using publicly available algorithms including miRwalk online prediction software (http://mirwalk.umm.uni-heidelberg.de/) and TargetScan 7.2 (www.targetscan.org). The GO terms and KEGG pathway terms enriched in the predicted target genes were determined using DAVID Bioinformatics.

### Exosomal RNA isolation and miRNA measurement

miRNA was isolated from EVs using the miRNeasy Micro Kit (Qiagen, Hilden, Germany) following the manufacturer’s instructions. The concentration and quality of RNA were determined using a NanoDrop 2000 (Thermo Fisher Scientific, Wilmington, DE, USA). The mature miRNAs were detected by reverse transcription and quantitative RT-PCR reaction using the All-in-One miRNA qRT-PCR Detection Kit (GeneCopoeia, Rockville, MD, USA) according to the manufacturer’s instructions. The primer mix was obtained from GeneCopoeia (GeneCopoeia, Rockville, MD, USA). The miRNA expression data were normalized to the expression of miR-23a-3p, miR-26a-5p, and let-7a-5p.

### Statistical analysis

We present the experimental data as means ± SD. We performed one-way analysis of variance (ANOVA) for multiple groups and two-tailed unpaired *t* test when comparing the two groups using GraphPad Prism 8 (San Diego, CA, USA). The *P* values are shown in the figures.

## Supplementary Material

aay6721_SM.pdf

## SUPPLEMENTARY MATERIALS

Supplementary material for this article is available at http://advances.sciencemag.org/cgi/content/full/6/13/eaay6721/DC1 Supplementary Materials and Methods Fig. S1. White/beige adipogenic differentiation of HASCs. Fig. S2. Human adipogenesis PCR array. Fig. S3. UCP1 assay. Fig. S4. Immunohistochemistry of the grafts treated with P-EV and D-EV. Fig. S5. High-performance liquid chromatography. Fig. S6. Adipose tissue browning in inguinal white adipose tissue. Fig. S7. Overall schematic diagram for exosomes derived from human adipose stem cells during white/beige adipogenic differentiation for cell-free therapeutic systems. Table S1. The information of HASCs purchased from Cefo Bio Co. Ltd. Table S2. Sequences of RT-PCR primers.

## Appendix

View/request a protocol for this paper from *Bio-protocol*.
