# Supplementary material for: Cell reprogramming using extracellular vesicles from differentiating stem cells into white/beige adipocytes
Source: Sci Adv. 2020 Mar 25;6(13):eaay6721. doi: 10.1126/sciadv.aay6721 (PMC7096171; doi:10.1126/sciadv.aay6721)
Supplement: aay6721_SM.pdf [file aay6721_SM.pdf]

Science Advances
AAAS

advances.sciencemag.org/cgi/content/full/6/13/eaay6721/DC1

# Supplementary Materials for

## Cell reprogramming using extracellular vesicles from differentiating stem cells into white/beige adipocytes

Youn Jae Jung, Hark Kyun Kim, Yoonsuk Cho, Ji Suk Choi, Chang Hee Woo, Kyoung Soo Lee, Jae Hoon Sul, Chan Mi Lee, Jihoon Han, Jae Hyung Park, Dong-Gyu Jo*, Yong Woo Cho*

*Corresponding author. Email: jodg@skku.edu (D.-G.J.); ywcho7@hanyang.ac.kr (Y.W.C.)

Published 25 March 2020, *Sci. Adv.* **6**, eaay6721 (2020)
DOI: 10.1126/sciadv.aay6721

**This PDF file includes:**

Supplementary Materials and Methods
Fig. S1. White/beige adipogenic differentiation of HASCs.
Fig. S2. Human adipogenesis PCR array.
Fig. S3. UCP1 assay.
Fig. S4. Immunohistochemistry of the grafts treated with P-EV and D-EV.
Fig. S5. High-performance liquid chromatography.
Fig. S6. Adipose tissue browning in inguinal white adipose tissue.
Fig. S7. Overall schematic diagram for exosomes derived from human adipose stem cells during white/beige adipogenic differentiation for cell-free therapeutic systems.
Table S1. The information of HASCs purchased from Cefo Bio Co. Ltd.
Table S2. Sequences of RT-PCR primers.

## Supplementary Materials and Methods

**Real time and reverse transcription-polymerase chain reaction** We extracted total mRNA from human adipose-derived stem cells (HASCs), white adipocytes (WA), and beige adipocytes (BA) using RNeasy RNA extraction kit (Qiagen, Germany). We performed reverse transcription of 0.5 μg total RNA using $RT^2$ First Strand kit (Qiagen, Hilden, Germany), and performed real time PCR in duplicate with SYBR Green Master Mix (Qiagen). We calculated relative mRNA gene expressions using β–actin as a control and the $2^{-\Delta ct}$ method. We also performed RT-PCR analysis using ONE-STEP RT-PCR PreMix kit (iNtRON) according to the manufacturer's protocol. We used cDNA as a template for PCR analysis with primers specific for human beta actin (β-actin) and uncoupling protein 1 (UCP1). The final PCR products were stained with Loading STAR (DyneBio) and separated by electrophoresis in 1.5% agarose gels.

**UCP1 enzyme-linked immunosorbent assay (ELISA)** We determined the concentrations of UCP1 in HASCs, WA, BA, and EV treated HASCs by ELISA according to the manufacturer's recommendations (Elabscience, Houston, TX, USA).

**Human adipogenesis PCR array** A $RT^2$ Profiler™ PCR Array Human Adipogenesis was purchased from QIAGEN (PAHS-049Z (330231), Hilden, Germany). Total RNA was extracted from P-EV, D-EV, and BD-EV treated HASCs using an RNA-spin™ Total RNA extraction kit (iNtRON BIOTECHNOLOGY, Sungnam, Korea) and cDNA was synthesized from 1 μg of total RNA using an $RT^2$ First Strand Kit (QIAGEN). $RT^2$ SYBR Green qPCR Master mix (QIAGEN), RNase-free water, and cDNA were mixed and dispensed into each well of the $RT^2$ Profiler PCR array according to the manufacturer's protocols. qPCR data were analyzed using QIAGEN's website software (Data analysis center; https://geneglobe.qiagen.com/kr/analyze/)

**High-performance liquid chromatography (HPLC)** HPLC was equipped with a fluorescence detector, system controller, pump, and data integrator (YL 9100 HPLC system, Young Lin Instrument Co., Ltd., Korea). We carried out chromatic separations at room temperature on a Zorbax Eclipse Plus C18 column (Analytical 4.6 × 250 mm 5-Micron, Agilent, USA). The mobile phase consisted of (40:60, *v*/*v*) acetonitrile and 10 mM ammonium acetate buffer (pH 5.2) with a low rate of 1 mL/min. We detected peaks using the fluorescence detector set at excitation and emission wavelengths of 247 nm and 367 nm, respectively. To prepare standard solution, we dissolved 1 mg/mL of rosiglitazone in absolute ethanol solution. Then we diluted the solution to 1 μg/mL with triple-distilled water for appropriate detection. We prepared the medium the same way for beige adipogenic differentiation with $1 \times 10^9$ particles/mL of BD-EV.

**Mitochondria assay** We observed mitochondria in living cells in HASCs, white and beige adipocytes by CytoPainter Mitochondrial Staining Kit (ab112145, abcam, MA, USA). Cells were finally visualized with a light microscope (KI-2000, KOREA LABTECH CORPORATION, Korea).

**Figures**

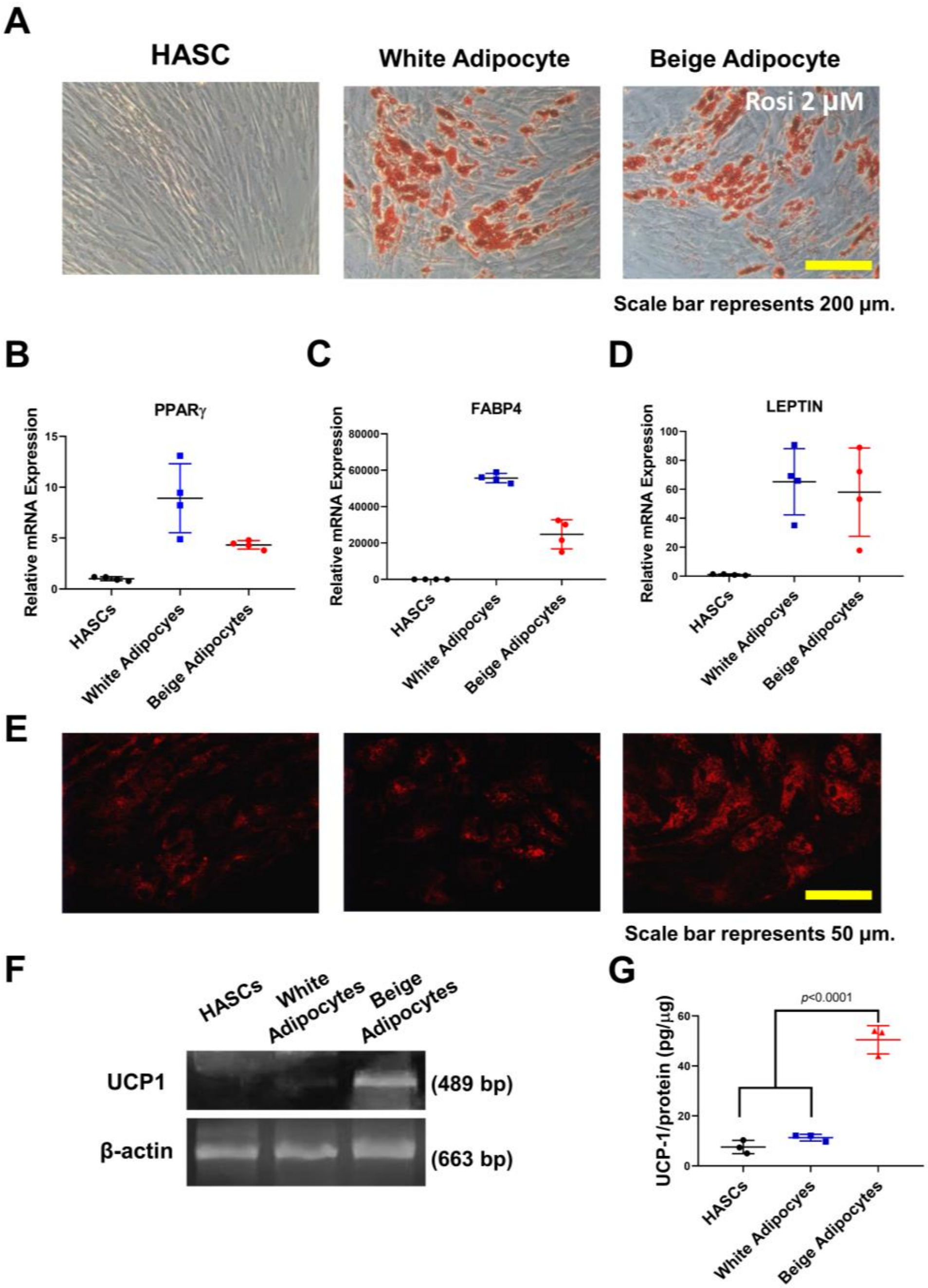


**Fig. S1. White/beige adipogenic differentiation of HASCs.** (**A**) HASCs maintained in white and beige adipogenic differentiation medium (DM) were analyzed by oil red O staining. HASCs were used as a negative control. Scale bars represent 200 μm. Real time polymerase chain reactions were performed for (**B**) PPARγ, (**C**) FABP4, and (**D**) LEPTIN expression in HASCs, white adipocytes and beige adipocytes. (**E**) Mitochondria were detected by mitochondrial staining assay in HASCs, white adipocytes, and beige adipocytes. (**F**) Reverse transcription polymerase chain reactions were performed for UCP1 mRNA expression in HASCs, WA, and BA. (**G**) UCP1 protein concentrations in HASCs, white adipocytes, and beige adipocytes were determined by ELISA ($p$<0.001 HASCs and white adipocytes vs. beige adipocytes, $n$=3).

**A**

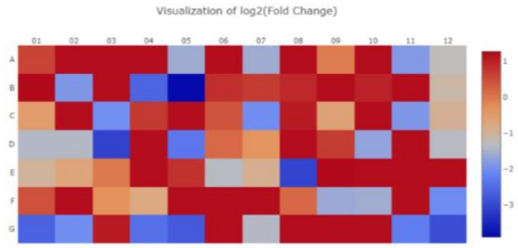


| Layout | 01 | 02 | 03 | 04 | 05 | 06 | 07 | 08 | 09 | 10 | 11 | 12 |
|---|---|---|---|---|---|---|---|---|---|---|---|---|
| A | ACACB 1.52 | ADIG 2.36 C | ADIPOQ 2.36 C | ADRB2 2.36 C | AGT -3.00 A | ANGPT2 2.36 C | AXIN1 -3.00 | BMP2 2.36 C | BMP4 -1.03 | BMP7 2.36 C | CCND1 -3.62 | CDK4 -2.45 |
| B | CDKN1A 2.42 | CDKN1B -3.69 A | CEBPA 2.36 C | CEBPB -6.08 A | CEBPD -14.87 A | CFD 1.81 | CREB1 1.63 | DDIT3 1.90 | DIO2 2.36 C | DKK1 1.98 | DLK1 2.36 C | E2F1 -2.15 |
| C | EGR2 -1.42 A | FABP4 2.36 C | FASN -4.01 | FGF1 1.65 | FGF10 2.36 C | FGF2 1.33 | FOXC2 -4.04 A | FOXO1 2.11 | GATA2 -1.54 | GATA3 2.36 C | HES1 -3.69 | INSR -1.92 A |
| D | IRS1 -2.66 | IRS2 -2.65 | JUN -8.54 A | KLF15 2.36 C | KLF2 -5.04 | KLF3 1.14 | KLF4 -1.28 | LEP 2.36 C | LIPE 1.63 | LMNA -3.26 | LPL 2.36 C | LRP5 -2.57 A |
| E | MAPK14 -2.02 | NCOA2 -1.62 A | NCOR2 -1.00 | NR0B2 2.36 C | NR1H3 1.75 | NRF1 -2.57 | PPARA -1.87 A | PPARD -8.36 A | PPARG 2.42 | PPARGC1A 2.36 C | PPARGC1B 2.36 C | PRDM16 2.36 C |
| F | RB1 1.37 | RETN 2.36 C | RUNX1T1 -1.25 | RXRA -1.70 A | SFRP1 2.36 C | SFRP5 2.36 C | SHH 2.36 C | SIRT1 1.15 | SIRT2 -3.08 A | SIRT3 -2.98 A | SLC2A4 2.36 C | SRC -4.10 A |
| G | SREBF1 -6.16 A | TAZ -4.04 A | TCF7L2 2.11 | TSC22D3 -5.40 A | TWIST1 -6.79 A | UCP1 2.36 C | VDR -2.63 A | WNT1 2.36 C | WNT10B 2.36 C | WNT3A 2.36 C | WNT5A -4.74 | WNT5B -7.59 A |

**B**

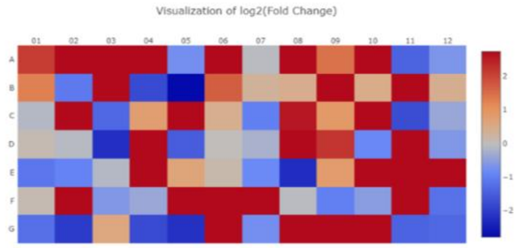


| Layout | 01 | 02 | 03 | 04 | 05 | 06 | 07 | 08 | 09 | 10 | 11 | 12 |
|---|---|---|---|---|---|---|---|---|---|---|---|---|
| A | ACACB 4.32 | ADIG 6.68 C | ADIPOQ 6.68 C | ADRB2 6.68 C | AGT -1.69 A | ANGPT2 6.68 C | AXIN1 -1.06 | BMP2 6.68 C | BMP4 2.75 | BMP7 6.68 C | CCND1 -2.68 | CDK4 -1.58 A |
| B | CDKN1A 2.41 | CDKN1B -2.13 A | CEBPA 6.68 C | CEBPB -3.48 A | CEBPD -6.96 A | CFD 3.23 | CREB1 1.23 A | DDIT3 1.36 | DIO2 6.68 C | DKK1 1.39 | DLK1 6.68 C | E2F1 1.32 |
| C | EGR2 -1.09 A | FABP4 6.68 C | FASN -2.58 A | FGF1 1.72 A | FGF10 6.68 C | FGF2 1.33 | FOXC2 -2.00 A | FOXO1 5.98 | GATA2 1.84 | GATA3 6.68 C | HES1 -3.43 A | INSR -1.33 A |
| D | IRS1 1.06 | IRS2 -1.07 | JUN -4.72 A | KLF15 6.68 C | KLF2 -2.91 | KLF3 1.00 | KLF4 -1.18 | LEP 6.68 C | LIPE 4.63 | LMNA -1.82 | LPL 6.68 C | LRP5 -1.55 A |
| E | MAPK14 -2.19 A | NCOA2 -1.93 A | NCOR2 -1.09 | NR0B2 6.68 C | NR1H3 1.56 | NRF1 1.10 | PPARA -1.80 A | PPARD -4.79 A | PPARG 1.78 A | PPARGC1A 6.68 C | PPARGC1B 6.68 C | PRDM16 6.68 C |
| F | RB1 1.06 A | RETN 6.68 C | RUNX1T1 -1.54 A | RXRA -1.31 A | SFRP1 6.68 C | SFRP5 6.68 C | SHH 6.68 C | SIRT1 -1.06 A | SIRT2 -2.00 A | SIRT3 -1.45 A | SLC2A4 6.68 C | SRC -2.31 A |
| G | SREBF1 -2.36 A | TAZ -4.14 A | TCF7L2 1.49 | TSC22D3 -3.48 A | TWIST1 -4.59 A | UCP1 6.68 C | VDR -1.69 A | WNT1 6.68 C | WNT10B 6.68 C | WNT3A 6.68 C | WNT5A -2.69 A | WNT5B -2.57 A |

**Fig. S2. Human adipogenesis PCR array.** Heat map and the fold change value of the (**A**) D-EV and (**B**) BD-EV treated HASCs compared to the P-EV treated group. Red blocks in heat map refer to relatively high expressed genes and blue blocks in heat map refer to relatively low expressed genes, respectively. These data were analyzed by RT$^2$ profiler PCR array for human adipogenesis.

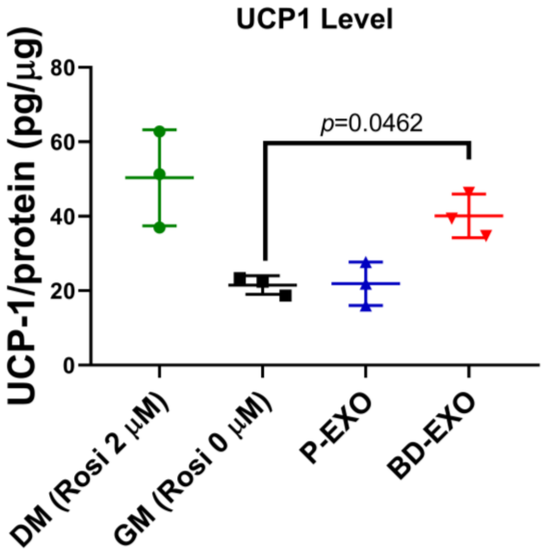


**Fig. S3. UCP1 assay.** UCP1 protein concentrations in HASCs after treatment with rosiglitazone (2 µM), P-EV ($1 \times 10^8$ exosomes/mL) and BD-EV ($1 \times 10^8$ exosomes/mL) for 2 weeks ($P$ = 0.0462 growth medium vs. BD-EXO $1 \times 10^8$).

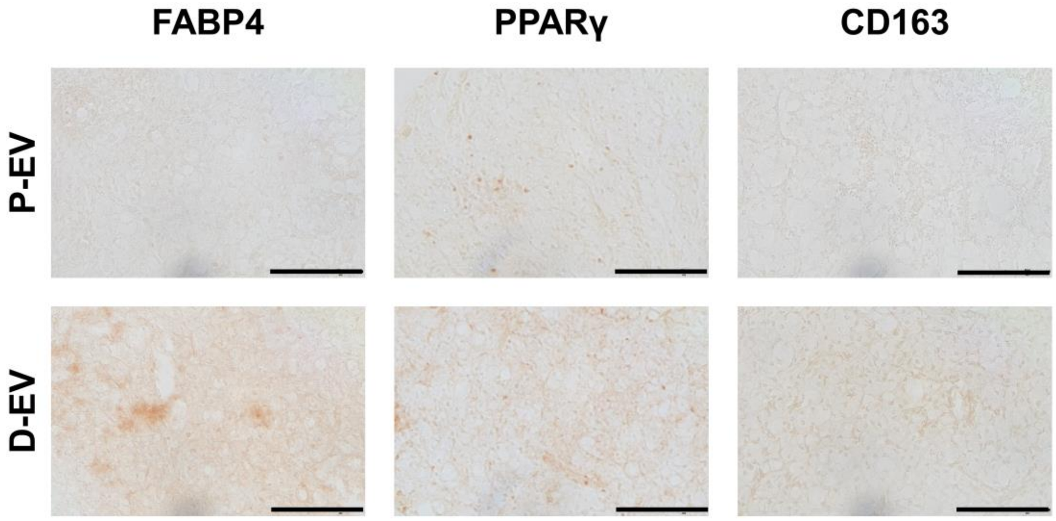


**Fig. S4. Immunohistochemistry of the grafts treated with P-EV and D-EV.** Immunostaining of FABP4, PPARγ, and CD163 in sections of P-EV and D-EV containing hydrogels. The scales bars represent 100 µm.

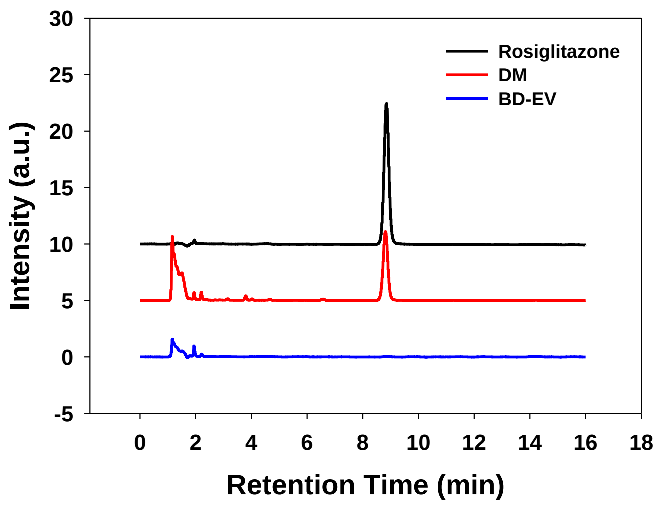


**Fig. S5. High-performance liquid chromatography.** Chromatograms of rosiglitazone (black line), differentiation medium (DM) containing rosiglitazone (red line), and BD-EV (blue line) analyzed by HPLC. All experiments were performed under optimized conditions.

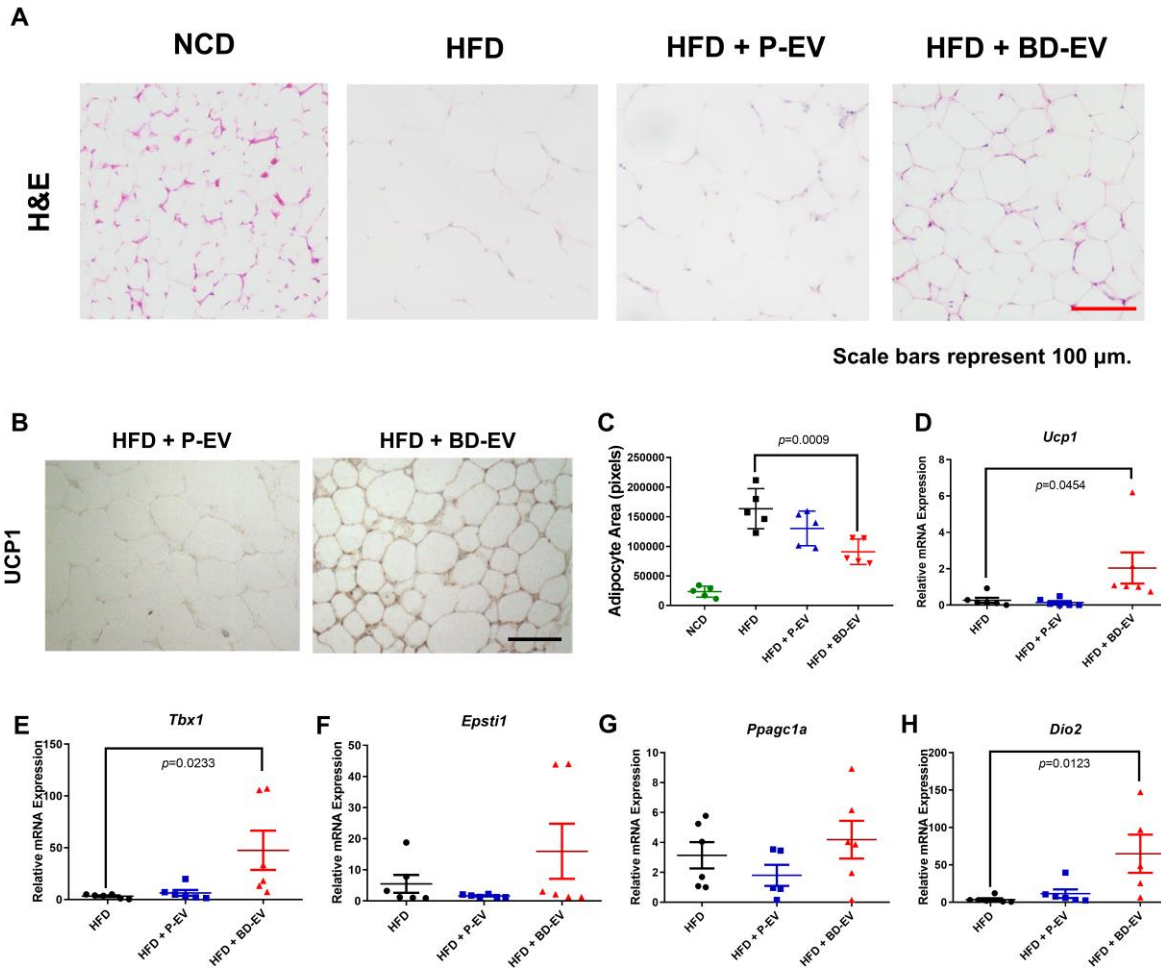


**Fig. S6. Adipose tissue browning in inguinal white adipose tissue.** (**A**) Representative images stained with H&E of iWAT from 16-week-old HFD-fed mice treated with PBS, P-EV, or BD-EV for 8 weeks. NCD-fed mice used as controls. (**B**) Immunohistochemistry *Ucp1* in subcutaneous adipose tissue in P-EV and BD-EV treated mice. The scale bar represents 100 µm. (**C**) Adipocyte areas of iWAT were measured using Image J (*n*=5 per group) the p values are shown in the figures. Treatment with BD-EV induces the expression of markers of brown adipocytes in iWAT. (**D-H**) mRNA levels of markers of brown and beige adipose tissue (*n*=6 per group) were highly expressed in iWAT treated with BD-EV (Brown adipose tissue marker: *Ppargc1a*, *Dio2*, and *Ucp1*; Beige adipose marker: *Tbx1* and *Epsti1*). The *P* values are shown in the figures.

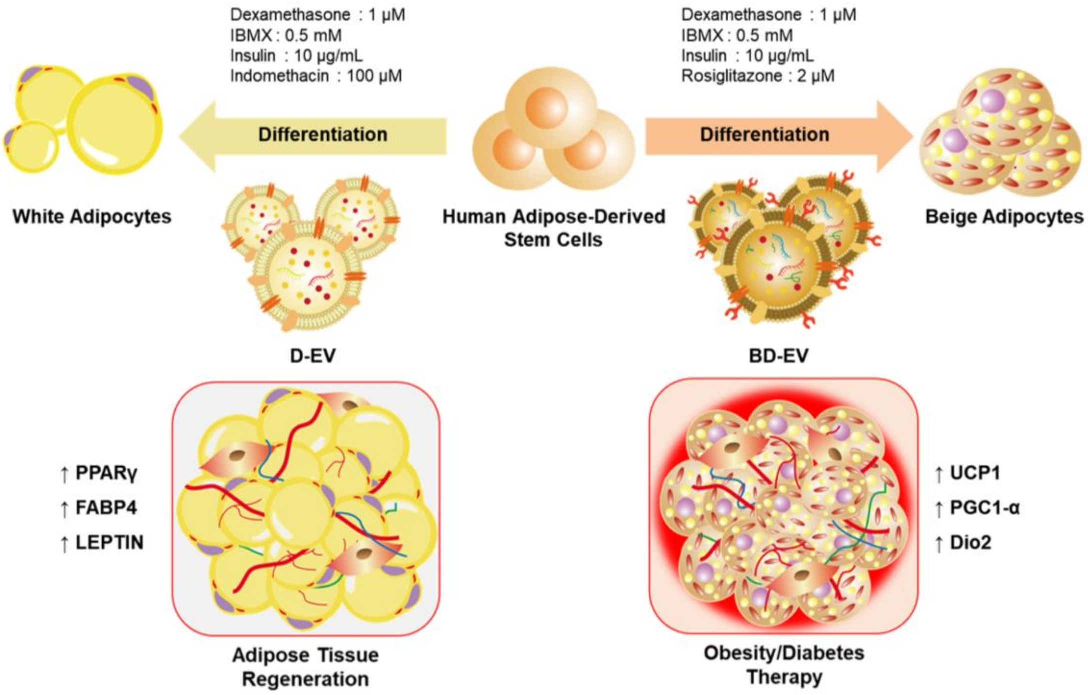


**Fig. S7. Overall schematic diagram for exosomes derived from human adipose stem cells during white/beige adipogenic differentiation for cell-free therapeutic systems.** EVs from HASCs during white and beige adipogenic differentiation (D-EV and BD-EV, respectively) contain various factors for differentiation of stem cells into white/beige adipocytes and promote adipogenesis *in vitro* and *in vivo*. D-EV promoted adipose tissue regeneration *in vivo* at 4 weeks after injections, resulting in elevated PPARγ, FABP4 and Leptin expression. BD-EV prevented DIO by inducing adipose tissue browning. Genes related to adipose tissue browning (UCP1, PGC1-α, Dio2) were highly expressed in BD-EV-treated mice.

**Table S1. The information of HASCs purchased from Cefo Bio Co. Ltd.**

| Lot No. | Age | Gender | Fat Isolation Depot |
|---|---|---|---|
| 70E07-060 | 36 | Female | Buttocks |
| 70E21-062 | 38 | Female | Thighs |

**Table S2. Sequences of RT-PCR primers.**

| Gene | Forward and reverse primer sequences | Annealing temperature (ºC) | Product size (bp) | Origin | Gene bank accession no. |
|---|---|---|---|---|---|
| β-actin | 5’-TGACGGGGTCACCCACACTGTGCCATCTA-3’<br>5’-CTAGAAGCATTTGVGGTGGACGATGGAGGG-3’ | 60 | 663 | Human | NM001101 |
| UCP1 | 5’-TGGAATAGCGGCGTGGCTTG-3’<br>5’-CTCATCAGATTGGGAGTAG-3’ | 60 | 489 | Human | NM021833.4 |
